# *Fusarium elaeidis* Causes Stem and Root Rot on *Alocasia longiloba* in South China

**DOI:** 10.3390/pathogens10111395

**Published:** 2021-10-28

**Authors:** Yunxia Zhang, Chao Chen, Jingpeng Zhao, Cantian Chen, Jieying Lin, Ruvishika S. Jayawardena, Meimei Xiang, Ishara S. Manawasinghe, Chunping You

**Affiliations:** 1 Innovative Institute for Plant Health, Zhongkai University of Agriculture and Engineering, Guangzhou 510225, China; zhangyunxia@zhku.edu.cn (Y.Z.); Chenchao_cc98@163.com (C.C.); jingpeng-zhao@yahoo.com (J.Z.); 18825187974@163.com (C.C.); linjieying178@163.com (J.L.); mm_xiang@163.com (M.X.); 2 Center of Excellence in Fungal Research, Mae Fah Luang University, Chiang Rai 57100, Thailand; ruvishikajay@mfu.ac.th; 3 School of Science, Mae Fah Luang University, Chiang Rai 57100, Thailand

**Keywords:** *Fusarium* rot, first report, ornamental plants, pathogenicity, *Nectriaceae*

## Abstract

*Alocasia longiloba* is a popular ornamental plant in China, however pests and diseases associated with *A. longiloba* reduce the ornamental value of this plant. From 2016 to 2021, stem and root rot has been observed on *A. longiloba* in Guangdong Province, China. Once the disease became severe, plants wilted and died. A fungus was isolated from the diseased stem and identified as *Fusarium elaeidis* using both morphological characteristics and molecular analysis of DNA-directed RNA polymerase II subunit (*rpb2*), translation elongation factor-1α (*tef1*) gene and β-tubulin (*tub2*) sequence data. The pathogenicity test showed the fungus was able to produce typical symptoms on *A. longiloba* similar to those observed in the field. The original pathogen was reisolated from inoculated plants fulfilling Koch’s postulates. This is the first report of *Fusarium elaeidis* causing stem rot on *A. longiloba*. These results will provide a baseline to identify and control diseases associated with *A. longiloba*.

## 1. Introduction

The ornamental plant industry has become a high demanding industry with the increased interest in modern architecture. The values of ornamental plants are based on the appearance of the plant. However, they are susceptible to several diseases including wilts, rots and leaf spots which reduce the economical values of the plants [1]. In addition, the ornamental plant industry is the main global source of planting material exchanges. Thus, it is possible to introduce a new pathogen to a new locality via these planting materials which can affect already established plants as well [2] Therefore, the identification and characterisation of phytopathogenic genera on ornamental plants are crucial.

Four *Alocasia* species are grown in China: *A. cucullata*, *A. hainanica*, *A. longiloba* and *A. macrorrhiza*. *Alocasia longiloba* is a perennial herb belonging to the *Araceae* (L). It is native to tropical Asia and in China this plant is mainly distributed in Hainan, Yunnan and Guangdong provinces. Among these *A. longiloba* is widely cultivated in Guangdong Province as an ornamental plant due to its distinctive ‘elephant ear’ foliage. There are several diseases and pests associated with *Alocasia* spp., including fungal diseases [3,4]. *Alternaria alocasiae* and *Ceratocystis fimbriata* have been reported to cause leaf spots [4] and leaf blight [5], respectively, on *A. macrorrhiza* in China [4]. In addition, anthracnose of *A. macrorrhiza* caused by *Colletotrichum karstii* [3], root rot on *Alocasia* sp. caused by *Pythium* sp. and *Rhizoctonia solani* in Florida, USA [6] are among reported diseases. Most of these studies were based on both morphological and phylogenetic aspects [3]. However, so far there are no records of fungal diseases on *A. longiloba* [7]. 

*Fusarium* is one of the well-known phytopathogenic genera that belong to the *Nectriaceae* [8,9]. In addition, they are also saprobes, endophytes, soil-borne or can be isolated from water [10]. *Fusarium* species are cosmopolitan, and the traditional classification of these fungi was based on morphology which has led to controversial arguments for many years. Species belonging to *F. solani* species complex were transferred to the *Neocosmospora* [11,12], however, this was not accepted by O’Donnell et al. 2020 [13] who suggested that the *F. solani* species complex should remain as a species complex within the *Fusarium* [13]. Species delineation in *Fusarium* is based on morphological characters and molecular phylogeny [14]. In the *Fusarium* taxonomy and molecular phylogeny, the rDNA fragments; the nuclear ribosomal internal transcribed spacer (ITS) and large subunit (LSU) are uninformative in species-level identification [15,16]. However, the *tef1* is highly informative at the species level [16]. In addition, DNA-directed RNA polymerase II subunit RPB1 (*rpb1*) and *rpb2* are also informative for species identification in *Fusarium* [14,17].

During 2016–2021, a new stem and rot rot disease was observed on *A. longiloba* in Guangzhou City (Guangdong Province, China). This study aims to identify the causal organism of this disease. Based on both morphological and molecular approaches, the pathogen was identified as *Fusarium elaeidis*. The pathogenicity of this species was confirmed on potted *A. longiloba* plants.

## 2. Results

### 2.1. Field Symptoms

**Figure 1:**
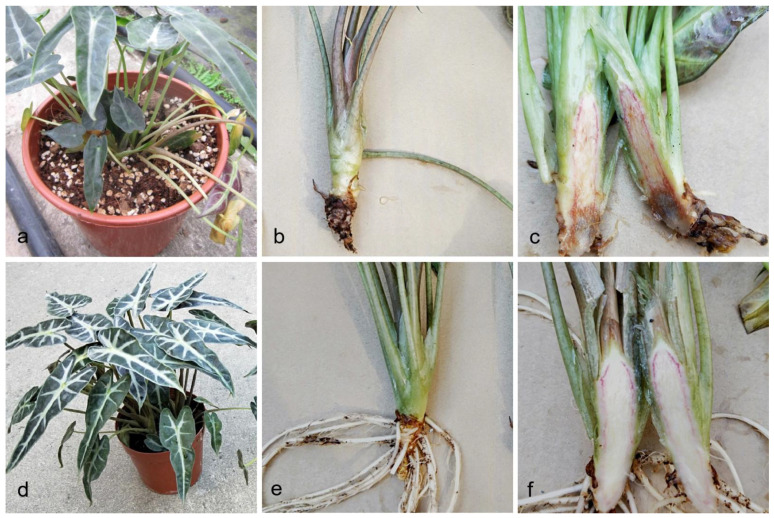
Field symptoms of stem and root rot on *Alocasia longiloba*. (**a**–**c**) Field symptoms of stem and root rot on *A. longiloba*; (**d**–**f**) Healthy plants of *A. longiloba* in the field.

In the field, a one pot usually contains three to four plants. In these pots one or two plants showed wilt symptoms. The disease begins with a progressive leaf yellowing, followed by wilting, until the whole plant collapsed at the end (Figure 1a). Roots of diseased plants became black and rotted (Figure 1b). When diseased plants were cut open longitudinally the inside of the stems appeared brown (Figure 1c). Disease incidence was as high as 10% in some nurseries in Guangzhou City.

### 2.2. Phylogenetic Analyses and Species Identification

**Figure 2:**
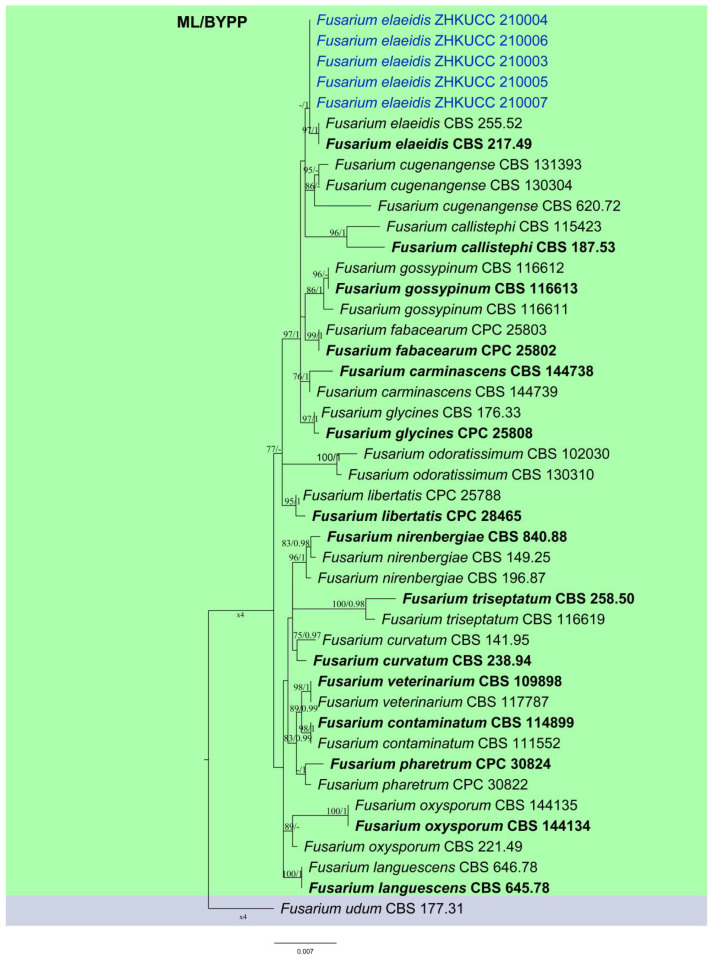
Phylogenetic tree of the *Fusarium* based on maximum likelihood and MrBayes of *rpb2*, *tef1* and *tub2* gene sequences. The ML bootstrap support values above 75% and BYPP higher than 0.90 are shown as ML/BYPP at each node. Some branches were shortened to fit them to the page. Ex-type isolates are bold, the isolates in the study are marked in blue.

The combined sequence data set of *rpb2*, *tef1*, and *tub2*, comprised five *Fusarium* isolates from this study and 38 reference sequences of *Fusarium* from Lomboard et al. [18]. The tree was rooted with *Fusarium udum* (CBS 177.31). The tree topology of the maximum likelihood (ML) analysis was similar to the Bayesian posterior probability analysis (BYPP). The best scoring RAxML tree with a final likelihood value of −4431.098940 is presented (Figure 2). The matrix had 150 distinct alignment patterns, with 0.42% undetermined characters or gaps.

Estimated base frequencies were as follows: A = 0.257323, C = 0.268459, G = 0.237268, T = 0.236950; substitution rates AC = 1.805852, AG = 3.992536, AT = 0.764421, CG = 1.200643, CT = 8.476617, GT = 1.000000; gamma distribution shape parameter α = 1.082532. In the phylogenetic tree, isolates from *A. longiloba* were grouped with *F. elaeidis* strains with 97% ML bootstrap support and 1.00 BYPP.

### 2.3. Taxonomy

**Figure 3:**
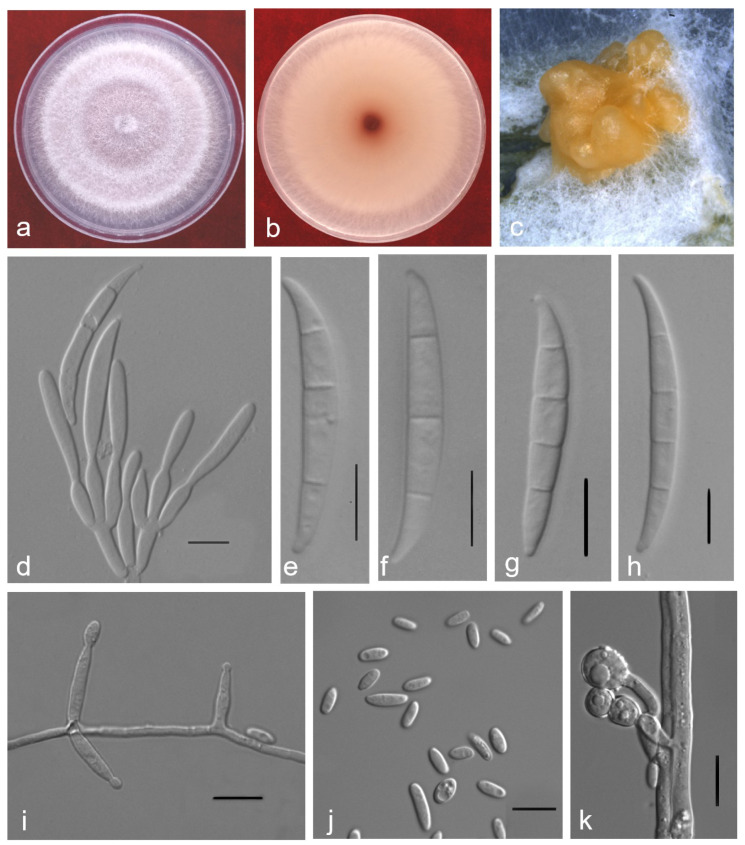
*Fusarium elaeidis* (ZHKUCC 21-0006). (**a**) surface of colony on PDA at 25 °C after three days; (**b**) reverse of colony on CLA at 25 °C after seven days; (**c**) sporodochia on carnation leaves; (**d**) sporodochial conidiophores and phialides; (**e**–**h**) macroconidia; (**i**) Aerial conidiophores phialides; (**j**) microconidia; (**k**) chlamydospores. Scale bars: d–k = 10 μm.

*Fusarium elaeidis* L. Lombard & Crous, Persoonia 41: 23 (2018) (Figure 3).

IF 826838, 

MycoBank MB826838 

Pathogenic on *Alocasia longiloba* Miq. *Sexual morph*: Not observed. *Asexual morph*: The colour of sporodochia was orange on the surface of carnation leaves. *Conidiophores* 8–27 × 2–5 μm ($\bar{x}$ 13 × 4 μm, n = 50) irregular, branched; sporodochial phialides doliiform to ampulliform. *Macroconidia* (1–) 3–4 (–5)-septa, 3-septate 30–50 × 3–7 μm ($\bar{x}$ 39 × 5 μm, n = 50), 4-septate 35–55 × 3–6 μm ($\bar{x}$ 46 × 4 μm, n = 50), hyaline, straight to slightly curved. *Apical cell* tapered and curved. *Basal cell* was notched or foot-shaped. *Conidiophore* 7–26 × 2–5 μm ($\bar{x}$ 13 × 3 μm, n = 50) phialides, on the aerial mycelium cylindrical, monophialidic. *Microconidia* 6–10 × 2–4 μm ($\bar{x}$ 8 × 3 μm, n = 50) produced in false heads on short phialides, hyaline with zero to one septum, ellipsoidal, reniform, or oval. *Chlamydospores* 6–15 × 4–11 μm ($\bar{x}$ 9 × 8 μm, n = 50), formed solitary, in pairs or chains, either terminal or intercalary in hyphae. *Culture characteristics*: Colonial growth rate was 6–7 mm on PDA at 25 °C per day. The colony surface was white, flat, and floccose with a regular margin. In reverse colony white at first and turned pink in the centre at the end. Sporodochia produced on carnation leaf agar (CLA) after 7 days.

*Material examined:* CHINA, Guangdong Province, Guangzhou, isolated from diseased stems of *A. longiloba* Miq., from November 2016 to April 2021, YX Zhang and CP You, dried cultures ZHKU 21-0003–21-0007; living culture ZHKUCC 21-0003–21-0007.

*Notes*: Isolates obtained from this study formed a well-supported clade with the representative strains of *Fusarium elaeidis* (CBS 217.49; Ex-type strain and CBS 255.52). Most of the morphological characteristics in this study were similar to the original description of *Fusarium elaeidis* by Lombard et al. [18]. However, the isolates obtained in this study differed by 3-septate and 4-septate of macroconidia. Moreover, in this study isolates produce larger macroconidia than those of the original description, and no polyphialides were observed in this study. The base pairs of both *rpb2* and *tub2* in this study are same as that of *F. elaeidis* (strain no?). Only three base pairs of *tef1* (totally 616 bp) were different with that of *F. elaeidis* (?) in Lombord et al. study [18]. Therefore, we identified our strains as *F. elaeidis* based on both morphology and molecular phylogeny.

### 2.4. Pathogenicity Test

**Figure 4:**
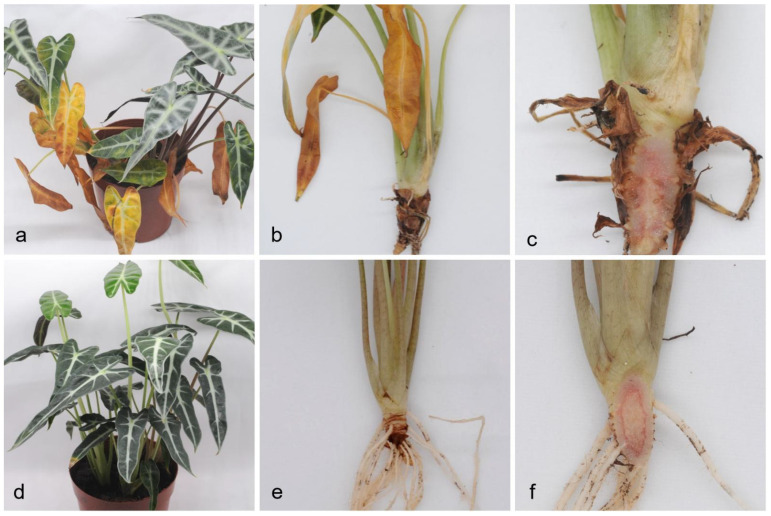
Inoculation assays of stem and root rot on *Alocasia longiloba* (inoculated with ZHKUCC 21-0005). (**a**–**c**) Symptom of stem and root rot after 15 days of inoculation; (**d**–**f**) Control plants of *A. longilob* after 15 days of inoculation.

The pathogenicity of isolates ZHKU 21-0005 and ZHKU 21-0006 was determined by inoculating healthy *A. longiloba* with mycelium suspension. Leaves of inoculated plants turned yellow after ten days (Figure 4a). Root and stem showed black and started to rot after fifteen days of inoculation (Figure 4b,c). The root density of inoculated plants was lower compared to healthy plants. Symptoms on the inoculated plants were similar to those on diseased plants in the field. None of the control plants developed symptoms (Figure 4d–f). From the diseased plants, the pathogen was reisolated. The colonies and morphological characteristics of isolates from inoculated plants were the same as those from the field.

## 3. Discussion

*Fusarium* is an important genus including large numbers of phytopathogenic species. Therefore, correct species identification is important for disease diagnosing and control. Twenty monophyletic species complexes have been reported in *Fusarium* [18]. The morphological species of *F. oxysporum* was recognized as a species complex because of its high level of phylogenetic diversity [18,19,20,21]. Laurence et al. [21] divided 17 independent evolutionary lineages into two phylogenetic species using genealogical concordance phylogenetic species recognition (GCPSR) criteria. Moreover, Lombard et al. [18] identified fifteen cryptic taxa using five gene regions in the *Fusarium*. Multi-genes analysis, such as ITS, IGS rDNA (the nuclear ribosomal intergenic spacer region), mating-type genes (*mat1-1-1*α-box), *tef1* and *tub2*, are used to delineate species in *Fusarium* and beyond species level within the *F. oxysporum* species complex [2,20,22,23,24]. In this study, we combined morphological characteristics with DNA sequence analysis of *rpb2*, *tef1* and *tub2* genes to ensure species identification accurately. 

*Fusarium oxysporum* species complex is a well-established and diverse complex in this genus. It can infect around 150 plant species [10], including vegetables, fruits, and ornamental plants, and cause severe diseases. Among these *F. oxysporum* f. sp. *cubense* which is the causal agent of banana wilt is one of the well-know and destructive disease on banana worldwide [25]. *Fusarium* *fabacearum* infect Malay apple plants (*Syzygium malaccensis*) and caused tree death in Brazil in 2017 [25]. On ornamentals, the disease caused by *F. oxysporum* cause wilt, crown rot and root rot. Moreover, these hosts included several valuable ornamental plants, such as *Cymbidium* spp., *Chrysanthemum* spp., and *Gladiolus* spp. [1]. *Fusarium* wilt is a destructive disease in cyclamen production. This disease was the first outbreak in Germany and then quickly spread to all European production regions resulting in a huge effect on the cyclamen industry [2,26]. *Fusarium* wilt on *Cymbidium* spp. is a well-known disease that leads to stem rot and plant death [27,28]. *Fusarium oxysporum* can infect various cultivars of *Cymbidium* and hybrid *Cymbidium*. The disease incidence was up to 30% in some plantations in Guangdong, China [28]. Over 30 ornamental plant genera have been recorded as hosts of *F. oxysporum* [1]. In the present study, we isolated and identified *F. elaeidis* from *A. longiloba* causing stem and root rot. 

*Fusarium oxysporum* f. sp. *elaeidis* was upgraded to the species level as *Fusarium elaeidis* by Lombard et al. [18]. In that study, they resolved 15 cryptic taxa which were named as *forma specialis* in *Fusarium oxysporum* [18]. Because of this, almost all the previous reports on this species are under *Fusarium oxysporum* f. sp. *elaeidis*. This species has been reported to cause *Fusarium* wilt in oil palm worldwide [29]. This pathogen is a soilborne fungus and difficult to control [30]. However, this is the first report of stem and root rot on *A. longiloba* caused by *F. elaeidis*. Therefore, further studies are necessary to understand the host range of this pathogen. *Fusarium* is a well-established genus with a larger number of forms and races. Given the same weight as other respective fields in mycology, identification of pathogenic species is the most critical step in plant pathology [31,32]. Moreover, in plant pathology, this identification is necessary to go beyond the species level. However, for most of the phytopathogenic fungal genera except like *Fusarium*, defining beyond species levels are not practised [32]. Even though lower-level ranking is well defined in *Fusarium,* in recent studies, some of these lower levels are upgraded into species level. Therefore, it is necessary to conclude how to delineate each species and define lower levels in *Fusarium.*

## 4. Materials and Methods

### 4.1. Sample Collection

A stem rot disease was observed on *A. longiloba* in a nursery in Guangzhou City (Guangdong Province, China) from 2016–2021. Diseased plants showed yellow leaves, rotted stems and wilting. Twenty plants from different greenhouses were collected in 2016, 2019 and 2021, respectively. Samples were placed in sterile, transparent plastic bags. Relevant photographs were taken on-site and samples were taken to the laboratory for further studies. In addition to that disease symptoms, sampling time and diseases severity were recorded at the time of sample collection.

### 4.2. Fungal Isolation and Purification 

Infected stems from diseased plants were first washed with running tap water to remove debris. Then the samples were cut into 0.5 × 0.5 cm sections including both healthy and diseased tissues. Those cuttings were surface sterilized by immersion in 75% ethanol for 10 s, 2.5% NaOCl for 40 s, and rinsed in sterile water three times. After that samples were dried in sterilized tissue paper. Dried cuttings were placed onto potato dextrose agar (PDA) and incubated at 25 °C. Pure cultures were obtained after three times hyphal tip isolations. In total four isolates were obtained. The cultures were deposited in the Culture Collection of Zhongkai University of Agriculture and Engineering (ZHKUCC 21-0003, ZHKUCC 21-0004, ZHKUCC 21-0005, ZHKUCC 21-0006, ZHKUCC 21-0007).

### 4.3. DNA Extraction, PCR Amplification and Sequencing

The genomic DNA of four isolates was extracted using a DNA rapid Extraction Kit (Aidlab Biotechnologies Co., Ltd, Beijing, China). A portion of DNA-directed RNA polymerase II subunit (*rpb2*), the translation elongation factor-1α gene (*tef1*) and the β-tubulin genes (*tub2*) were amplified and sequenced. The *tef1* region was amplified using primer pair EF-1H and EF-2T [33]. The *tub2* region was amplified using primer pair T1 and CYLTUB1R [22,34]. The *rpb2* region was amplified using primer pair 5f2 and 7cr [35,36]. PCR reactions were conducted in 25 μL volumes containing 12.5 μL of 2× Easy Taq PCR SuperMix (TransGen Biotech, Beijing, China), 1 μL DNA, each of 5μM premier (1 μL), ddH2O (9.5 μL). PCR amplification was performed with an initial denaturation step of 94 °C for 5 min, followed by 35 cycles of 94 °C for 40 s, annealing at 55 °C for 45 s, and extension at 72 °C for 45 s, and a final extension at 72 °C for ten min. The PCR products were sequenced by Guangzhou Tianyi Huiyuan Science and Technology Co. Ltd (Guangzhou, China). For sequencing in both directions with forward and reverse primers were used. The DNA sequence of *rpb2*, *tef1* and *tub2* were deposited in the GenBank (Table 1). 

### 4.4. Phylogenetic Analyses

Consensus sequences were derived from forward and reverse primer sequences for *rpb2*, *tef1* and *tub2*, and compared against the GenBank database (https://blast.ncbi.nlm.nih.gov/Blast.cgi, accessed on 18 June 2021). Relevant sequence data of *Fusarium* species were obtained from the National Center for Biotechnology Information (NCBI) following Lomboard et al. [18]. Sequences were aligned using MAFFT version 7. Sequences were aligned manually using BioEdit where necessary. Concatenated dataset of *rpb2*, *tef1,* and *tub2* were used for the phylogenetic analyses.

Phylogenetic analyses were conducted based on Maximum-likelihood in RAxML [37] and Bayesian analyses in MrBayes (v. 3.0b4) [38] The maximum likelihood analyses were conducted using RAxML-HPC2 on XSEDE (8.2.8) [39] in the CIPRES Science Gateway platform. The GTR+I+G evolution model was used with 1000 non-parametric bootstrapping iterations. Bayesian analysis was performed with six simultaneous Markov chains were run for 106 generations, sampling the trees at every 200th generation. From the 10,000 trees obtained, the first 2000 representing the burn-in phase were discarded. The remaining 8000 trees were used to calculate posterior probabilities (PPs) in a majority rule consensus tree. The final sequence alignment generated in this study was submitted to TreeBASE (https://treebase.org/treebase-web/home.html, accessed on 5 July 2021) under submission ID 28480.

### 4.5. Morphological Characterisation 

Pure cultures incubated on PDA for 7 days at 25 °C under a 12 h light-dark cycle were used for colony characteristics and colonial growth rates. Cultures grown on carnation leaves agar (CLA) for 7–10 days at 25 °C under a 12 h light-dark cycle were used for microscopic characters observation. Morphological characters were photographed using an ECLIPSE 80i microscope (Nikon, Tokyo, Japan) and measurements were taken using NIS-Elements BR 3.2. Measurements of spore length and width of 50 spores were taken. The mean values were calculated with Microsoft Excel. 

### 4.6. Pathogenicity Tests

Two representative isolates ZHKU21-0005 and ZHKU21-0006 were used to inoculate potted plants from the nursery. Representative isolates were incubated in PDA for three days to obtain mycelia. One gram of mycelium added into 100 mL sterilised water and blend to make into 3% mycelium suspension. Healthy plants were inoculated by pouring 50 mL of mycelium suspension into the substrate around the roots. Sterilised water (50 mL) was used as the control. For each treatment three replicates were used. All treated plants were maintained at 25 °C in a growth chamber. To fulfil Koch’s postulates, the fungus was re-isolated from inoculated plants showing typical disease symptoms and compared to the original isolate.

## Figures and Tables

**Table 1:** GenBank accession numbers of *Fusarium* strains used in phylogenetic analysis. The isolates obtained in this study are bold.

Species	Culture Accession	GenBank Accession Numbers		
		*rpb2*	*tef1*	*tub2*
*Fusarium callistephi*	CBS 187.53 ^T^	MH484875	MH484966	MH485057
	CBS 115423	MH484905	MH484996	MH485087
*F. carminascens*	CBS 144739 = CPC 25792	MH484934	MH485025	MH485116
	CBS 144738 = CPC 25800 ^T^	MH484937	MH485028	MH485119
*F. contaminatum*	CBS 111552	MH484900	MH484991	MH485082
	CBS 114899 ^T^	MH484901	MH484992	MH485083
*F. cugenangense*	CBS 620.72 = DSM 11271 = NRRL 36520	MH484879	MH484970	MH485061
	CBS 130304 = BBA 69050 = NRRL 25433	MH484921	MH485012	MH485103
	CBS 131393	MH484928	MH485019	MH485110
*F. curvatum*	CBS 238.94 = NRRL 26422 = PD 94/184 ^T^	MH484893	MH484984	MH485075
	CBS 141.95 = NRRL 36251 = PD 94/1518	MH484894	MH484985	MH485076
*F. elaeidis*	CBS 217.49 = NRRL 36358	MH484870	MH484961	MH485052
	CBS 255.52 = NRRL 36386	MH484874	MH484965	MH485056
	**ZHKUCC 21-0003**	**MZ439841**	**MZ325284**	**MZ439836**
	**ZHKUCC 21-0004**	**MZ439842**	**MZ325285**	**MZ439837**
	**ZHKUCC 21-0005**	**MZ439843**	**MZ325286**	**MZ439838**
	**ZHKUCC 21-0006**	**MZ439844**	**MZ325287**	**MZ439839**
	**ZHKUCC 21-0007**	**MZ439845**	**MZ325288**	**MZ439840**
*F. fabacearum*	CBS 144743 = CPC 25802 ^T^	MH484939	MH485030	MH485121
	CBS 144744 = CPC 25803	MH484940	MH485031	MH485122
*F. glycines*	CBS 176.33 = NRRL 36286	MH484868	MH484959	MH485050
	CBS 144746 = CPC 25808 ^T^	MH484942	MH485033	MH485124
*F. gossypinum*	CBS 116611	MH484907	MH484998	MH485089
	CBS 116612	MH484908	MH484999	MH485090
	CBS 116613 ^T^	MH484909	MH485000	MH485091
*F. languescens*	CBS 645.78 = NRRL 36531 ^T^	MH484880	MH484971	MH485062
	CBS 646.78 = NRRL 36532	MH484881	MH484972	MH485063
*F. libertatis*	CBS 144747 = CPC 25788	MH484933	MH485024	MH485115
	CBS 144749 = CPC 28465 ^T^	MH484944	MH485035	MH485126
*F. nirenbergiae*	CBS 840.88 ^T^	MH484887	MH484978	MH485069
	CBS 149.25 = NRRL 36261	MH484865	MH484956	MH485047
	CBS 196.87 = NRRL 26219	MH484886	MH484977	MH485068
*F. odoratissimum*	CBS 102030	MH484898	MH484989	MH485080
	CBS 130310 = NRRL 25603	MH484922	MH485013	MH485104
*F. oxysporum*	CBS 221.49 = IHEM 4508 = NRRL 22546	MH484872	MH484963	MH485054
	CBS 144134 ^ET^	MH484953	MH485044	MH485135
	CBS 144135	MH484954	MH485045	MH485136
*F. pharetrum*	CBS 144750 = CPC 30822	MH484951	MH485042	MH485133
	CBS 144751 = CPC 30824 ^T^	MH484952	MH485043	MH485134
*F. triseptatum*	CBS 258.50 = NRRL 36389 ^T^	MH484873	MH484964	MH485055
	CBS 116619	MH484910	MH485001	MH485092
*F. veterinarium*	CBS 109898 = NRRL 36153 ^T^	MH484899	MH484990	MH485081
	CBS 117787	MH484912	MH485003	MH485094

CBS: Westerdijk Fungal Biodivesity Institute (WIFB), Utrecht, The Netherlands. ZHKUCC: Zhongkai University of Agriculture and Engineering culture collection. Sequences produced in this study are shown in bold. Type sequnces are given as ^T^.

## Data Availability

The data presented in this study are given in Table 1.
